# The *Rickettsia conorii* Adr1 Interacts with the C-Terminus of Human Vitronectin in a Salt-Sensitive Manner

**DOI:** 10.3389/fcimb.2017.00061

**Published:** 2017-03-01

**Authors:** Abigail I. Fish, Sean P. Riley, Birendra Singh, Kristian Riesbeck, Juan J. Martinez

**Affiliations:** ^1^Vector-Borne Diseases Laboratories, Department of Pathobiological Sciences, Louisiana State University School of Veterinary MedicineBaton Rouge, LA, USA; ^2^Clinical Microbiology, Department of Translational Medicine, Lund UniversityMalmö, Sweden

**Keywords:** Adr1, complement, *Rickettsia*, Spotted-Fever Group *Rickettsia*, serum resistance, vitronectin

## Abstract

Spotted fever group (SFG) *Rickettsia* species are inoculated into the mammalian bloodstream by hematophagous arthropods. Once in the bloodstream and during dissemination, the survival of these pathogens is dependent upon the ability of these bacteria to evade serum-borne host defenses until a proper cellular host is reached. *Rickettsia conorii* expresses an outer membrane protein, Adr1, which binds the complement inhibitory protein vitronectin to promote resistance to the anti-bacterial effects of the terminal complement complex. Adr1 is predicted to consist of 8 transmembrane beta sheets that form a membrane-spanning barrel with 4 peptide loops exposed to the extracellular environment. We previously demonstrated that Adr1 derivatives containing either loop 3 or 4 are sufficient to bind Vn and mediate resistance to serum killing when expressed at the outer-membrane of *E. coli*. By expressing *R. conorii* Adr1 on the surface of non-pathogenic *E. coli*, we demonstrate that the interaction between Adr1 and vitronectin is salt-sensitive and cannot be interrupted by addition of heparin. Additionally, we utilized vitroenctin-derived peptides to map the minimal Adr1/vitronectin interaction to the C-terminal region of vitronectin. Furthermore, we demonstrate that specific charged amino acid residues located within loops 3 and 4 of Adr1 are critical for mediating resistance to complement-mediated killing. Interestingly, Adr1 mutants that were no longer sufficient to mediate resistance to serum killing still retained the ability to bind to Vn, suggesting that Adr1-Vn interactions responsible for resistance to serum killing are more complex than originally hypothesized. In summary, elucidation of the mechanisms governing Adr1-Vn binding will be useful to specifically target this protein-protein interaction for therapeutic intervention.

## Introduction

Spotted Fever group (SFG) Rickettsia are Gram-negative, obligate intracellular bacteria that are transmitted to a mammalian host when an arthropod vector takes a blood meal (Riley et al., 2012). Members of the SFG include the human pathogens *R. conorii* and *R. rickettsii*, the etiologic agents of Mediterranean Spotted Fever and Rocky Mountain Spotted Fever, respectively. Upon inoculation into a host, the bacteria can spread throughout the body via the bloodstream and parasitize cells of many origins, including endothelial cells, monocytes, macrophages, and hepatocytes (Walker and Gear, 1985; Walker et al., 1994, 1999; Walker, 1997; Riley et al., 2016). Infection of endothelial cells can lead to disruption of the endothelial lining and increased fluid leakage which causes the characteristic macropapular rash, thus the name Spotted Fever (Walker and Ismail, 2008). If left untreated, infections can lead to severe manifestations of the disease such as renal failure, non-cardiogenic pulmonary edema, interstitial pneumonia, and ultimately death (Walker and Ismail, 2008). When rickettsial infection is properly diagnosed, treatment is generally successful; however, misdiagnosis is common due to initial non-descript flu-like symptoms, which leads to increased morbidity and mortality (Chan et al., 2009; Riley et al., 2014).

Because of the obligate intracellular nature of SFG rickettsia, the bacteria must bind to and invade a host cell in order to survive and proliferate (Walker and Ismail, 2008). However, during arthropod feeding and rickettsia dissemination, the bacteria are extracellula and as a result are exposed to the hostile environment of the mammalian bloodstream (Riley et al., 2014). While outside the safety of the host cell cytosol, the bacteria are exposed to the bactericidal effects of the host's complement system and survival of these bacteria are dependent upon their ability to evade killing until a proper cellular host is reached (Riley et al., 2012).

The complement system contributes to both the innate and adaptive immune system, and serves as the first line of defense against invading organisms. Complement is composed of fluid-phase and membrane bound proteins that can be activated through three different mechanisms (Singh et al., 2010a). The classical, the lectin, and the alternative pathways are initiated by antibodies or various proteins that recognize structures on the surface of a microbe and the pathways converge at the formation of C3 convertase to form the common lytic pathway (Blom et al., 2009). This results in the deposition of membrane attack complex (MAC) proteins C5b through C9 on the surface of the pathogen and accumulation of these proteins leads to formation of a lytic pore in the membrane which causes osmotic cell lysis (Singh et al., 2010b). Other functions of complement include increased opsonization of the pathogen by binding of C3b components to the surface of the microbe and stimulation of inflammatory responses with the proteins C3a, and C5a (Blom et al., 2009).

The complement cascade must be kept under strict control, bercause activation can result in significant inflammation and can attack both foreign molecules as well as a self components. As such, the host utilizes a series of regulatory proteins which include Factor I, Factor H, C4-binding protein, vitronectin, and clusterin (Singh et al., 2010b). These proteins associate with the surface of host cells in order to control and block complement activation (Hallstrom et al., 2015). Many bacterial pathogens, including *Moraxella catarrhalis, Neisseria meningitidis, Streptococcus pyogenes, S. pneumoniae, Staphylococcus aureus*, and *Pseudomonas aeruginosa* have evolved mechanisms to utilize these host regulatory proteins to their advantage, thereby protecting themselves from complement-mediated attacks (Liang et al., 1997; Singh et al., 2010a; Griffiths et al., 2011; Voss et al., 2013; Riley et al., 2014; Hallstrom et al., 2015).

A previous report demonstrated that *R. conorii* is inherently resistant to complement-mediated killing when exposed to human serum (Chan et al., 2011). This information led to the discovery of a protein that is expressed on the surface of *R. conorii*, termed Adr1, that contributes to the serum resistance phenotype by binding the multifunctional human glycoprotein vitronectin (Riley et al., 2014). Vitronectin binds to complement proteins C5b-C7 and C9 to inhibit deposition of the MAC on the bacterial surface (Singh et al., 2011). The *R. conorii* vitronectin-binding protein, Adr1, is a conserved outer membrane protein that is predicted to contain 8 trans-membrane beta sheets that form a membrane spanning barrel, as well as four connecting beta strands termed “loops” that protrude into the extracellular environment (Vogt and Schulz, 1999; Riley et al., 2014). Our lab previously demonstrated that two of these domains, loops 3 and 4, were sufficient to interact with vitronectin and thus, mediate resistance to serum killing when Adr1 proteins containing either loop 3 or loop 4 were expressed in a serum-sensitive strain of *E. coli* (Riley et al., 2014). Interestingly, homologs of Adr1 are present in every sequenced rickettsial species to date and the deduced amino acid sequences of loops 3 and 4 are almost 100% conserved among SFG rickettsial species (Riley et al., 2014), suggesting that resistance to serum mediated killing mediated by Adr1 may be a widespread virulence attribute in this class of obligate intracellular pathogens.

In the present study, we further analyzed the interaction between Adr1 and vitronectin with the intention of understanding in detail the mechanisms of interaction. We demonstrated that the interaction of Adr1 with vitronectin is sensitive to increasing salt concentrations, and not competitively inhibited by increasing concentrations of heparin. Using various truncated, recombinant vitronectin peptides, we also demonstrate that the Adr1-vitronectin interaction maps to a region located in the C-terminal domain of vitronectin. Furthermore, we utilized site-directed mutagenesis to determine the specific amino acids located within loops 3 and 4 of Adr1 that are critical in mediating resistance to complement-mediated killing in serum.

## Materials and methods

### Construction of mutants in Adr1 by PCR

Plasmids pJP01-L3 and pJP01-L4 as previously described (Riley et al., 2014) were utilized as a template for quick-change site-directed mutagenesis PCR to mutate individual lysine residues in each loop to alanine residues. Briefly, plasmids pJP01-L3 and pJP01-L4 contain the gene for an Adr1 derivative with only intact loop 3 or loop 4, respectively. All other loops have been reduced to the bare minimum amino acids necessary to maintain structural integrity of the protein (Riley et al., 2014). Primers for mutations can be found in Supplemental Table 1. In each case, parental DNA was digested using DpnI leaving mutant plasmid, which was then transformed into MaxEfficiency DH5-alpha-T1 (Life Technologies) and sequenced. Mutants were constructed for single amino acid substitutions as well as multiple and multiple, sequential amino acid substitutions for all 6 lysine residues in pJP01-L3 and pJP01-L4. Amino acid mutations are designated in **Figure 3**.

### Bacterial strains and culture conditions

*E. coli* BL21 (DE3) (pJP01, pJP01-L3, pJP01-L4, pET22b, pJP01-L3 mutants, and pJP01-L4 mutants) were cultured in Luria-Bertani (LB) broth at 37°C with 50 μg/mL of carbenicillin overnight. Bacteria were then diluted 1:10 into fresh media and grown to an OD_600_ between 0.5 and 1.0. Protein expression was induced with 0.6 mM IPTG (Isopropyl β-D-1-thiogalactopyranoside) and cultures were grown for 4 h at 37°C.

### Antibodies

Rabbit Anti-Adr1 peptide polyclonal antibodies (pAb) were utilized for immunoblot analysis of Adr1-expressing *E. coli* as previously described (Riley et al., 2014). Anti-*E. coli* RNA polymerase monoclonal IgG3 antibody (Ab) was purchased from Affinity Bioreagents. Rabbit IgG anti-human vitronectin pAb was purchased from Complement Technology and sheep anti-human vitronectin IgG was from AbD Serotec. Mouse IgG anti-Histidine tag pAb directly conjugated to horseradish peroxidase (HRP) was purchased from GenScript. Both donkey anti-mouse IRDye 680 IgG and donkey anti-rabbit IRDye 800 IgG were purchased from LiCOR biosciences. HRP-conjugated goat anti-rabbit IgG was from Sigma and donkey anti-sheep IgG was purchased from Thermo-Fisher.

### Vitronectin binding assay

To evaluate vitronectin binding, *E. coli* BL21 (DE3) containing plasmids pJP01, pET22b, or mutants were grown as described previously. Approximately 1 × 10^6^ colony forming units (cfu) of each construct was washed with PBS and resuspended in 200 μL PBS containing 25% normal human serum (NHS) pooled from 5 healthy individuals (Innovative Research). For analysis of vitronectin binding in the presence of NaCl or heparin, NaCl was purchased from Fisher Chemical and Heparin Sodium Salt was purchased from MP Biomedicals, LLC. The Adr1 and mutant expressing bacteria were resuspended in 100 μL PBS containing increasing concentrations of either NaCl (0, 0.25, 0.50, or 1.0 M) or heparin (0, 1, 2, 4, 8, 12, 16, 20, or 500 nM). One hundred microliter of 50% NHS was then added to the heparin/*E.coli* or NaCl/*E.coli* solution for final NHS concentration of 25%. For analysis of binding of vitronectin peptides, 1 × 10^6^ cfu Adr1 expressing *E. coli* were resuspended in a 100 μL of a 5 μM solution of each indicated purified recombinant peptide that had been expressed and isolated from HEK293T cells (Singh et al., 2010a). For analysis of vitronectin binding to *R. conorii*, a live, frozen stock of *R. conorii* was allowed to come to room temperature and resuspended in 50 μL of a heparin or salt solution at concentrations previously mentioned and 50 μL of 50% NHS was added for a final NHS concentration of 25%. To assess differences in multimeric and monomeric vitronectin binding to mutant expressing *E. coli*, multimeric, and monomeric vitronectin were purchased from Innovative Research. Roughly 1 × 10^6^ cfu of Adr1 and mutant expressing bacteria were washed and resuspended in 25 μL of PBS. Twenty five *microliter* of 50 ng/mL of monomeric or multimeric vitronectin was then added for a final concentration of 25 ng/mL vitronectin. Adr1 expressing *E. coli* were incubated on ice when bacteria were resuspended in NHS whereas bacteria resuspended in monomeric, multimeric, or recombinant peptide vitronectin were incubated at room temperature. *R. conorii* were also incubated at room temperature. All samples were allowed to bind for 1 h with gentle agitation, bacteria were washed 3 times in PBS and vitronectin binding was determined by SDS-PAGE and western immunoblotting using rabbit anti-human vitronectin or sheep anti-human vitronectin as primary antibody and goat anti-rabbit IgG HRP or donkey anti-sheep IgG HRP as secondary antibody.

### Serum resistance assay

NHS pooled from 5 healthy individuals (NHS) (Innovative Research) was stored at −80°C as aliquots until use. *E. coli* BL21 (DE3) containing pJP01-L3, pJP01-L4, pET22b, or loop 3 and loop 4 mutants were grown as described above. Approximately 1 × 10^6^ bacteria were then washed with PBS and resuspended in either 200 μL of PBS or 200 μL PBS containing 10% NHS. Bacteria were incubated at 37°C for 15 min with agitation and then serially diluted in PBS and plated on LB-Agar plates. After overnight incubation, colony-forming units (cfu) were evaluated. Experiments were performed in triplicate with a minimum of 3 replicates for each experiment. Data is presented as the cfu of bacteria in PBS/cfu of bacteria in 10% NHS after 15 min of incubation and plotted on a logarithmic scale. Expression of Adr1 or Adr1 mutant proteins was verified by western immunoblotting using rabbit anti-Adr1 or mouse anti-histidine tag antibodies as described above.

### Bacterial fractionation

Induced *E. coli* BL21 (DE3) cultures (250 mL) were pelleted, resuspended in 5 mL PBS containing 1x protease inhibitor cocktail and then fractionated to enrich for outer-membrane proteins as described (Hancock and Nikaido, 1978). Briefly, bacteria were twice lysed in a French Pressure cell (1,500 psi) and unbroken cells were cleared by centrifugation at 3,000 g for 15 min at 4°C. Sarkosyl was added to the resulting supernatant (total cell lysate) to a final concentration of 0.5% and incubated with rotation at room temperature for 10 min to extract inner membrane proteins. Outer membrane proteins were pelleted by ultracentrifugation for 1 h at 100,000 g and resuspended in 2x sample buffer. Total cell lysate, inner membrane fraction and outer membrane fraction were resolved by SDS-PAGE and stained with Coomassie blue for visualization of total protein content and analyzed by western immunoblotting with anti-Adr1 antibodies.

### Statistical analysis

Statistical Analysis was performed using a one-way ANOVA with a Neuman-Keuls *post hoc* test or a Kruskal-Walis one-way ANOVA test to compare differences between more than two groups as indicated. Differences were considered significant with a *p*-value less than or equal to 0.05 using Graph-Pad Prism version 5.0b (GraphPad Software).

## Results

### Vitronectin binding is significantly reduced by increasing concentrations of NaCl, but is not substantially inhibited by heparin

**Figure 1:**
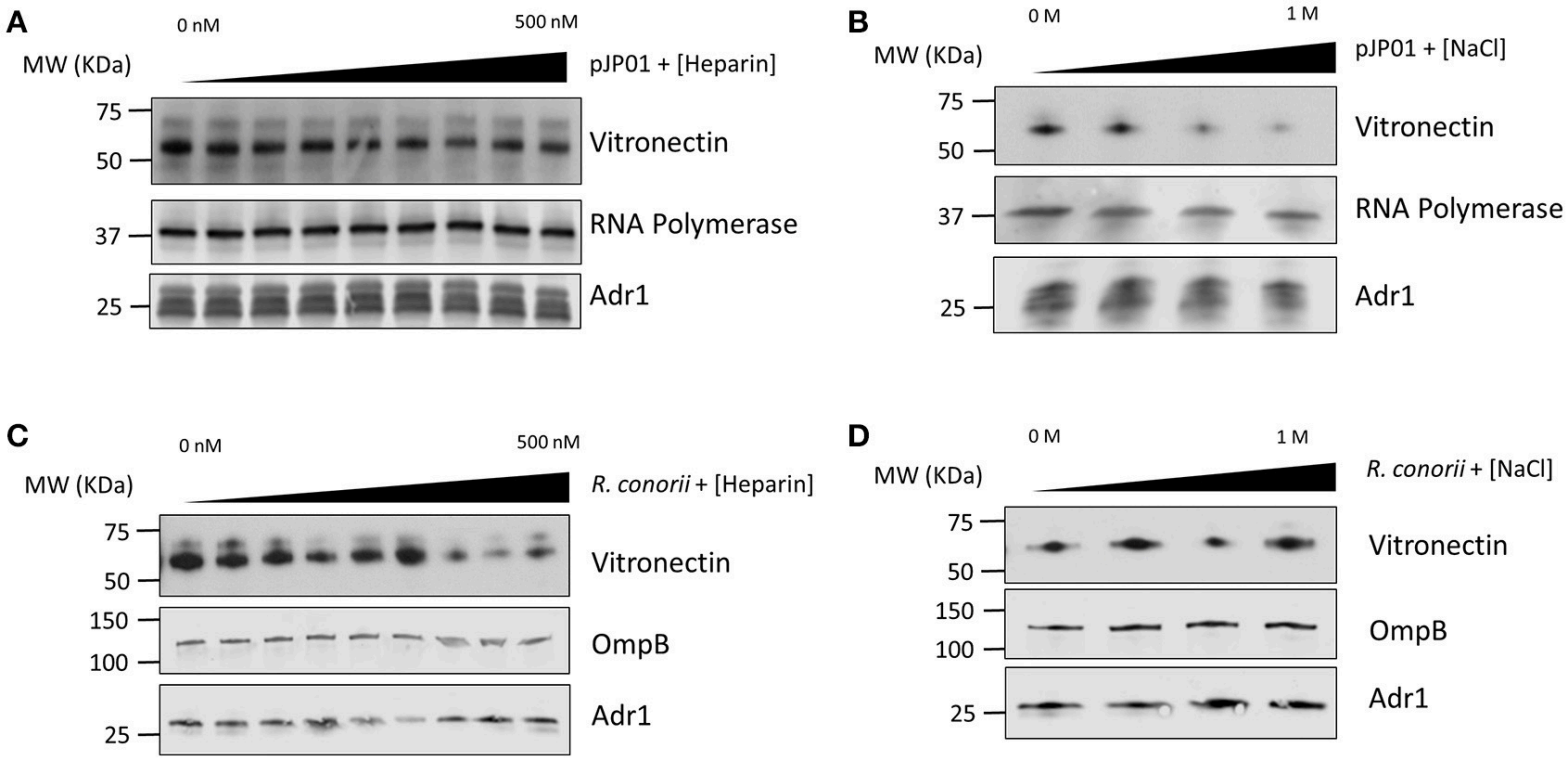
**Vitronectin binding to ***E. coli*** expressing Adr1 or ***R. conorii***. (A,B)** Western immunoblot analysis of vitronectin binding to *E. coli* expressing Adr1 in the presence of increasing concentrations of heparin (0–500 nM) or NaCl (0–1 M). Equal loading in each experiment was verified using an *E. coli* RNA polymerase antibody. Verification of Adr1 expression was validated using anti-Adr1. Data are representative of at least 3 independent experiments. **(C,D)** Western immunoblot analysis of vitronectin binding to *R. conorii* in the presence of increasing concentrations of heparin or NaCl. Equal loading in each experiment was verified using anti-OmpB and anti-Adr1 antibodies. Data are representative of 2 replicates.

Vitronectin contains many different functional regions that mediate its various roles in the human host (Singh et al., 2010a). Heparin-binding domains have been demonstrated to be involved in the interaction between vitronectin and Outer-membrane protein C (Opc) of *N. meningitis*, Ubiquitous surface protein A2 (UspA2) of *M. catarrhalis*, Lpd of *P. aeruginosa*, and several other pathogenic bacteria (Sa et al., 2010; Griffiths et al., 2011; Hallstrom et al., 2015). The charge of the individual amino acid residues within the binding domains of the outer membrane proteins of *S. pneumoniae* and *Haemophilus influenzae* have also been documented to play a role in binding (Hallstrom et al., 2009; Voss et al., 2013). Because of the high concentration of positively charged lysine residues located within loops 3 and 4 of Adr1, we hypothesized that these residues would play an important role in the protein-protein interaction. To determine if the *R. conorii* Adr1/human vitronectin interaction is a result of Adr1 interacting with the heparin binding domains or is an electrostatic interaction, Adr1 was expressed at the surface of *E. coli* and exposed to human serum in the presence of either increasing concentrations of heparin or NaCl. As the concentration of heparin in solution was increased 0–500 nM, which is approximately 3 times the concentration found in human plasma (Engelberg, 1961), there was no significant difference in ability of vitronectin to bind to Adr1 (Figure 1A and Supplemental Figure 1A). In contrast, increasing concentrations of NaCl in solution ranging from 0 to 1 M in the reaction, competitively inhibited the interaction between vitronectin and Adr1 (Figure 1B and Supplemental Figure 1B). Interestingly, when increasing concentrations of both NaCl or heparin were added to intact *R. conorii* cells in the presence of NHS, there was no significant difference in the ability of vitronectin to bind to *R. conorii* (Figures 1C,D and Supplemental Figures 1C,D). These data suggest that *R. conorii* and other pathogenic rickettsial species may possess additional factors that are sufficient to interact with vitronectin and may contribute to this phenotype. Nevertheless, this data suggests that the heparin-binding domains of vitronectin are not involved in the Adr1/vitronectin interaction, and that specific charged amino acid residues in loops 3 and 4 are likely critical in mediating this protein-protein interaction.

### Adr1 binds within the C-terminal region of vitronectin between amino acids 363 and 373

**Figure 2:**
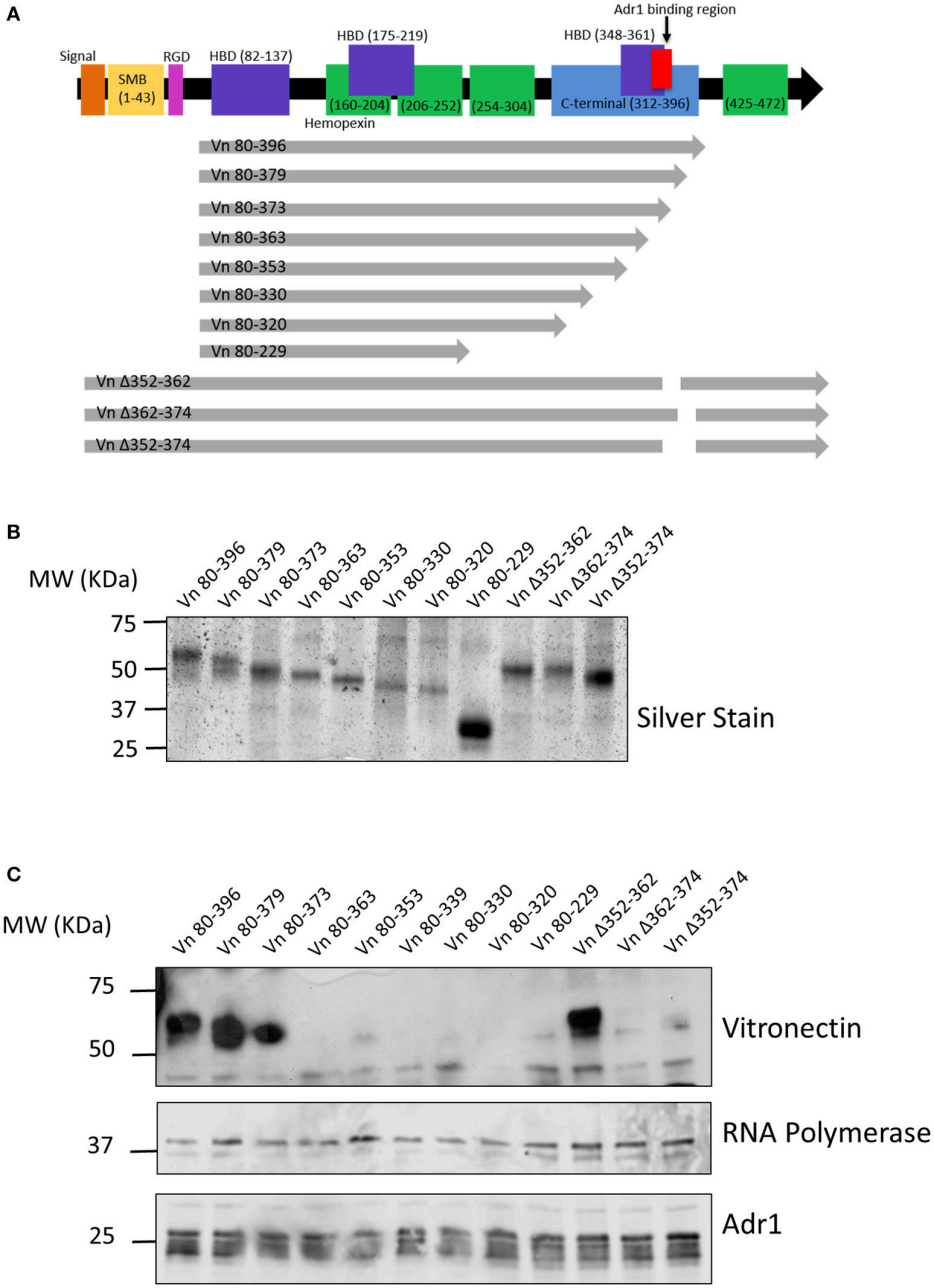
**Functional domains in vitronectin and peptides constructed for analysis of Adr1 binding region. (A)** Full length vitronectin with noted functional domains is depicted in the top black arrow with the constructed peptides depicted in gray arrows below. **(B)** Silverstain of vitronectin peptides utilized. The first 8 peptides begin at amino acid 80 and are progressively truncated at the C-terminus. The 3 full length peptides contain a deletion within the C-terminal region. **(C)** *E. coli* expressing Adr1 binds to the 3 longest vitronectin peptides and the full length peptide with a deletion at amino acids 352–362. Equal loading is demonstrated with anti-*E. coli* RNA polymerase and verification of expression of Adr1 is verified using anti-Adr1. Data are representative of at least 3 replicates.

We next wanted to identify the region(s) within vitronectin that mediate binding to Adr1. The C-terminal region (amino acids 312–396 depicted in Figure 2A) of vitronectin is well documented to mediate binding of serum resistance proteins from bacterial pathogens such as Protein E from *H. influenzae*, UspA2 from *M. catarrhalis*, and the PspC protein from *S. pneumoniae* (Singh et al., 2010a, 2011; Griffiths et al., 2011; Voss et al., 2013; Hallstrom et al., 2016). Because this region plays an important role in many interactions of both Gram-negative and Gram-positive bacteria, we hypothesized that Adr1 would also bind within the C-terminal region. To test this hypothesis, we performed vitronectin binding assays using a series of recombinant, truncated vitronectin peptides demonstrated in a silver stain in Figure 2B (Singh et al., 2010a; Su et al., 2013) and Adr1-expressing *E. coli* BL21(DE3). As shown in Figure 2C, peptides Vn 80–396, Vn 80–379, Vn 80–373, and Vn Δ352–362 were sufficient to bind to Adr1 expressed at the outer membrane of *E. coli*. Our data demonstrates that the region between amino acid 363 and 373 within the C-terminal region of vitronectin is involved in the binding of Adr1 and further demonstrate that Adr1-vitronectin interactions do not require heparin-binding domains.

### Serum survival of *E. coli* expressing Adr1 lysine to alanine mutants

**Figure 3:**
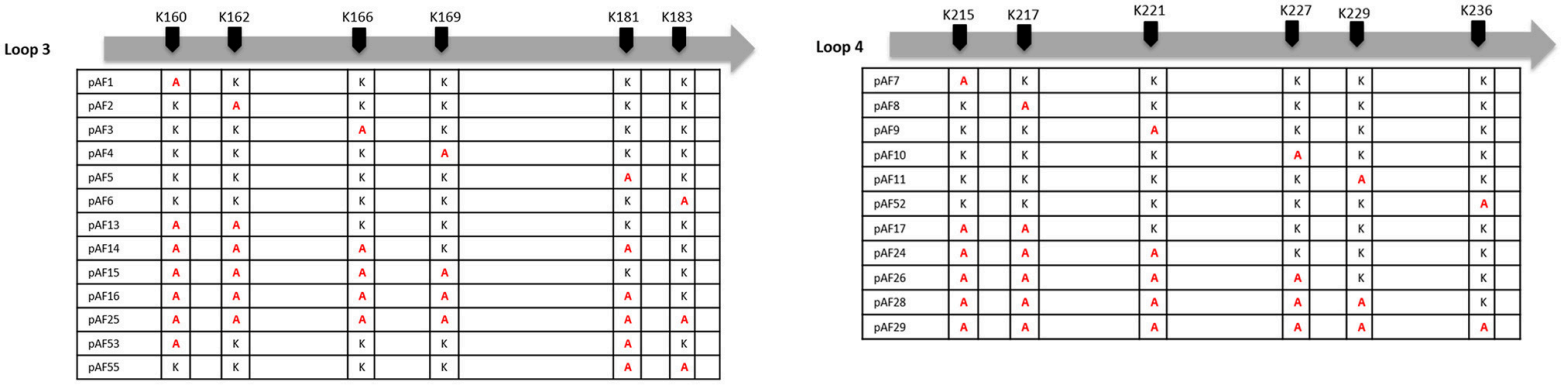
**Schematic representation of K-A substitutions in ***R. conorii*** Adr1 loops 3 and 4**. The position of each substituted lysine in loops 3 and 4 and the corresponding name of the plasmid is depicted above.

**Figure 4:**
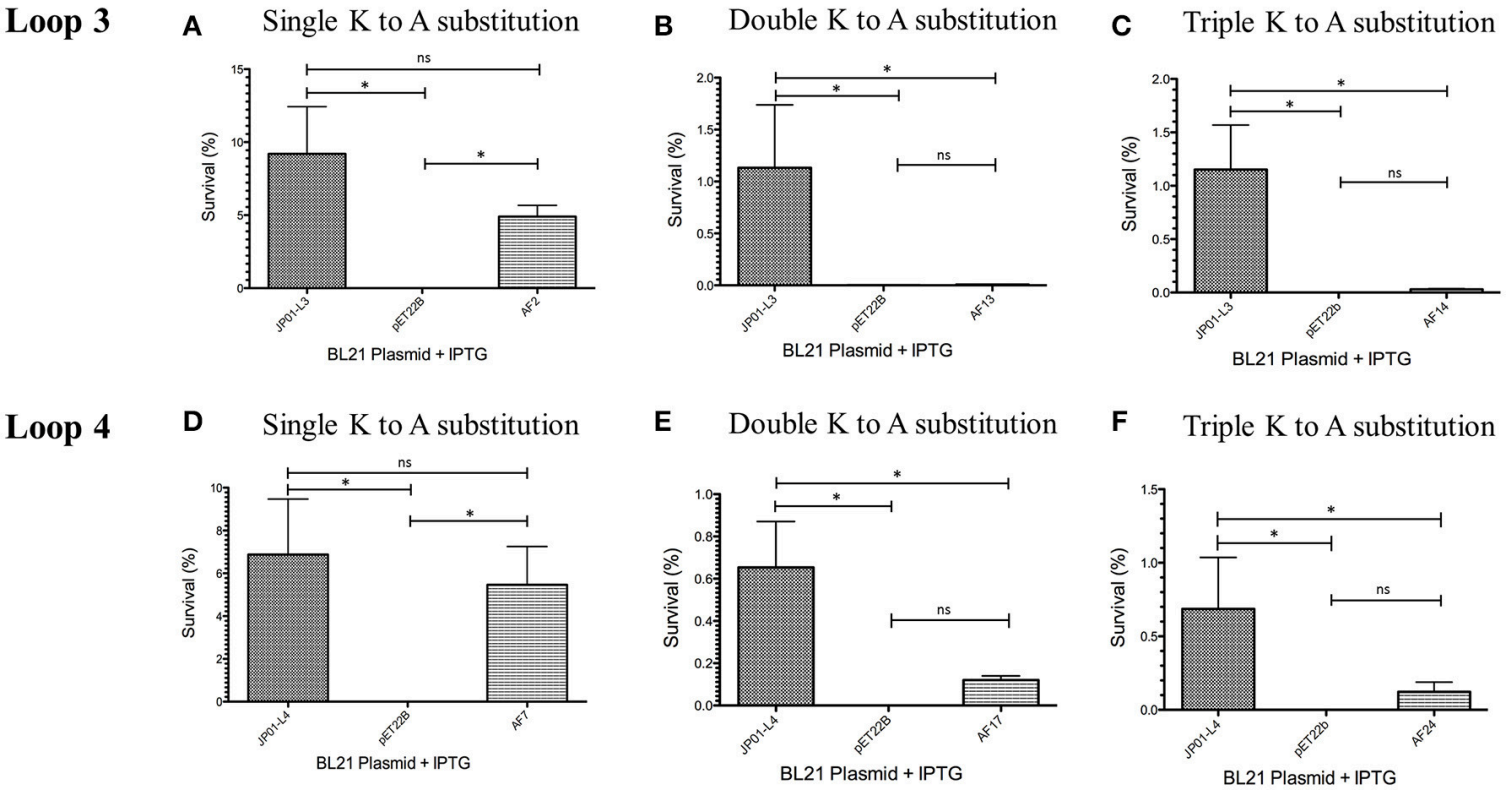
**Analysis of loop 3 and 4 mutant Adr1 serum resistance when expressed on the surface of ***E. coli*** and exposed to NHS. (A,D)** A single lysine to alanine substitution (AF2 and AF7). **(B,E)** A double lysine to alanine substitution at positions 1 and 2 (AF13 and AF17). **(C,F)** A triple amino acid substitution at positions 1, 2, and 3 (AF14 and AF24). Data are representative of at least 3 replicates. A one-way ANOVA with a Newman-Keuls *post hoc* test was performed. ^*^Represent a *p* ≤ 0.05 and is considered significant. ns represents no significance.

**Figure 5:**
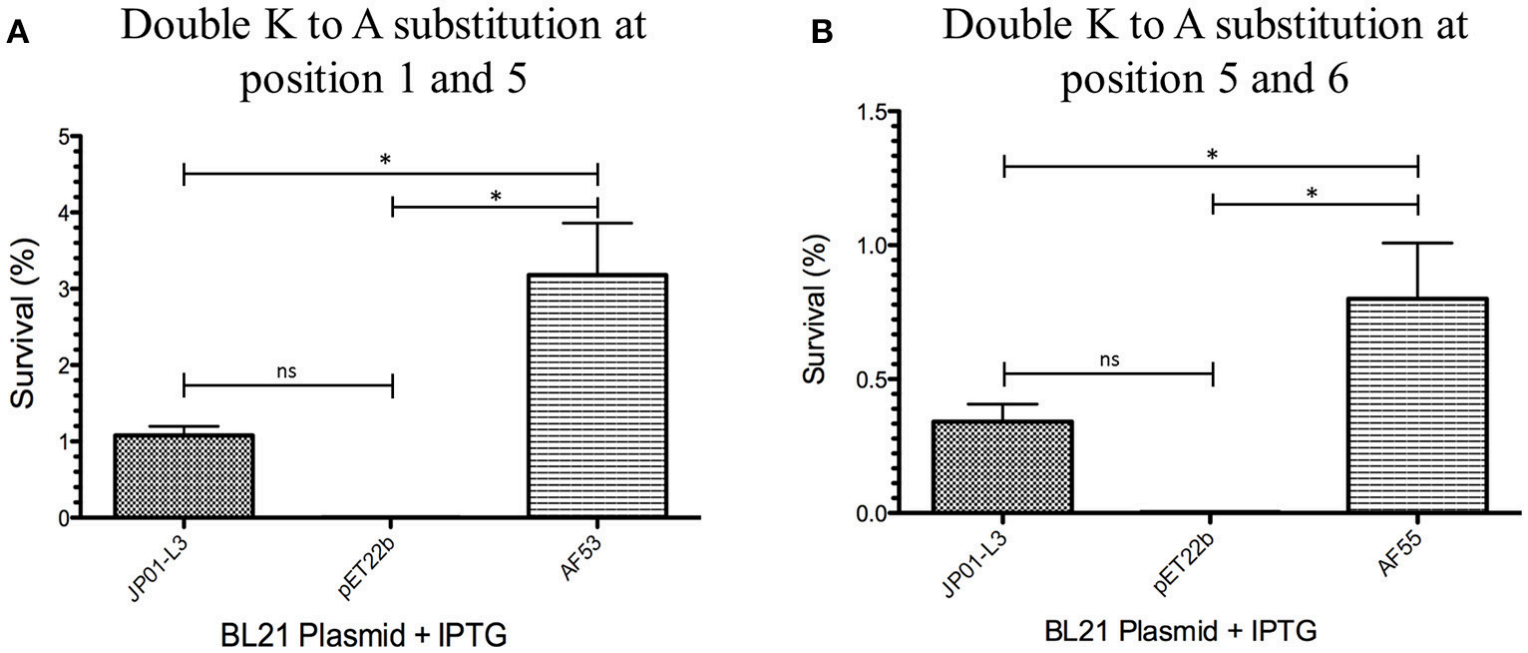
**Analysis double mutants of loop 3 Adr1 serum resistance when expressed on the surface of ***E. coli*** and exposed to NHS. (A)** A double lysine to alanine substitution at positions 1 and 5 (AF53). **(B)** A double lysine to alanine substitution at positions 5 and 6 (AF55). Data are representative of at least 3 replicates. A one-way ANOVA with a Newman-Keuls *post hoc* test was performed. ^*^Represent a *p* ≤ 0.05 and is considered significant. ns represents no significance.

We have previously demonstrated that extracellular Adr1 loops 3 and 4 are sufficient to both mediate resistance to complement-mediated killing and bind vitronectin (Riley et al., 2014). We, therefore, sought to identify residues within each loop that were responsible for the interaction. We utilized the previously constructed plasmids, pJP01-L3, and pJP01-L4 (Riley et al., 2014) which encode Adr1 proteins expressing only loops 3 or 4, respectively as templates for site-directed mutagenesis to substitute positively charged lysine residues to uncharged, non-polar alanine residues. A summary of the constructs used in these experiments is depicted in Figure 3. We initially verified expression of each construct by transforming a serum-sensitive strain of *E. coli* BL21 (DE3) with the indicated plasmid and inducing protein expression as described above. Western immunoblotting with anti-Adr1 antibodies confirmed the expression of each construct (Supplemental Figures 2, 3). Constructs pAF15, pAF16, pAF25, pAF28, and pAF29 did not express under any condition tested and were not further utilized. Because our data demonstrated that Adr1-Vn interactions are electrostatic in nature and that the Vn-interacting domains of Adr1 (loops 3 and 4) contain a high concentration of positively charged amino acids, we hypothesized that removal of one or more of the positive charge would decrease the ability of Adr1-expressing *E. coli* to survive when exposed to serum and to interact with Vn. To initially determine serum survival, each Adr1 mutant was expressed in *E. coli* and incubated in 10% NHS in PBS and PBS alone as a control. The cfu of bacteria exposed to NHS were quantified in comparison to the bacteria exposed to PBS and bacterial survival was expressed as the percent bacteria remaining after exposure to serum. As shown in Figures 4A,D, Adr1 mutants containing a single lysine to alanine substitution in any position within each loop remained resistant to serum killing. In contrast, when the first two or more positive charges to uncharged substitutions were made, the ability of *E. coli* expressing Adr1 mutant proteins to resist serum killing was significantly decreased (Figures 4B,C,E,F). These results suggest that the first two lysines in either loops 3 or 4 are critical to mediate resistance to serum mediated killing, but do not exclude the possibility that the net charge in either Adr1 loop contributes to the observed phenotype. We, therefore, designed experiments to distinguish between these two possibilities. Using the Adr1-loop 3 construct as a model, we created double lysine mutants at lysine positions 1 and 5 or at positions 5 and 6 (Figure 3), expressed these mutant Adr1 proteins in *E. coli*, and tested for their ability to survive in NHS. As shown in Figure 5, *E. coli* expressing the indicated lysine to alanine substitutions in Adr1 were able to survive when exposed to human serum. Taken together, these data demonstrate that the first two lysine residues in loop 3 are critical to mediate resistance to complement mediated killing, and suggests that these residues may play important roles in the interaction with vitronectin.

### Binding of multimeric vitronectin is correlated with survival of Adr1-expressing bacteria

**Figure 6:**
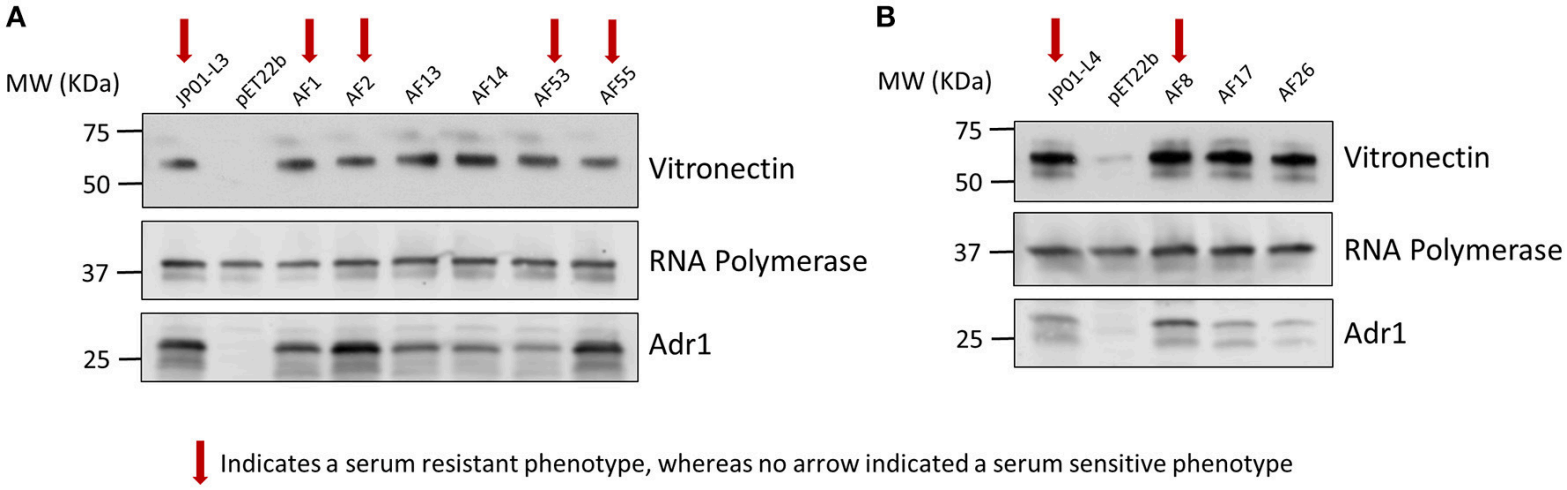
**Serum resistance or sensitivity does not correlate with the ability to bind vitronectin in serum. (A,B)** Western immunoblot analysis of vitronectin in serum binding to *E. coli* expressing loop 3 or loop 4 Adr1 mutants with both serum resistant and serum-sensitive phenotypes. Equal loading was verified using anti-*E. coli* RNA polymerase and Adr1 expression was verified using anti-Adr1. Data are representative of at least 3 replicates. Arrows depict Adr1 constructs that confer resistance to serum killing.

We next sought to verify that the serum-resistant phenotypes are correlated with the ability of Adr1 to bind vitronectin. We hypothesized that *E. coli* expressing Adr1 mutants that survived serum killing were sufficient to bind vitronectin, while *E. coli* expressing Adr1 mutants that did not survive in serum were either unable to bind or are greatly impaired in their ability to bind vitronectin. To test this hypothesis, we transformed *E. coli* BL21(DE3) with the constructs for serum resistant Adr1 loop 3 proteins harboring a single amino acid substitution construct, representing serum resistant phenotypes (pAF1 and pAF2), the double amino acid constructs at positions 1 and 5 (pAF53) and at position 5 and 6 (pAF55). Plasmids encoding for serum-sensitive phenotypes were also transformed, and include double substitution at positions 1 and 2 construct (pAF13) and triple substitution at positions 1, 2, and 3 construct (pAF14), representing the serum sensitive phenotype. Adr1 loop 4 mutant proteins containing a single amino acid substitution (pAF8, serum resistant) and the double and triple mutants (pAF17 and pAF26, serum-sensitive) were also expressed in *E. coli*. We initially verified the expression of Adr1 mutants at the outer membrane of *E. coli* when expressed under these conditions (Supplemental Figure 4). These bacteria were incubated with NHS, and analyzed for their abilities to interact with human Vn via a Western immunoblot analysis. Surprisingly, *E. coli* expressing either serum-resistant or serum-sensitive Adr1 mutant constructs were able to bind Vn in serum (Figures 6A,B).

**Figure 7:**
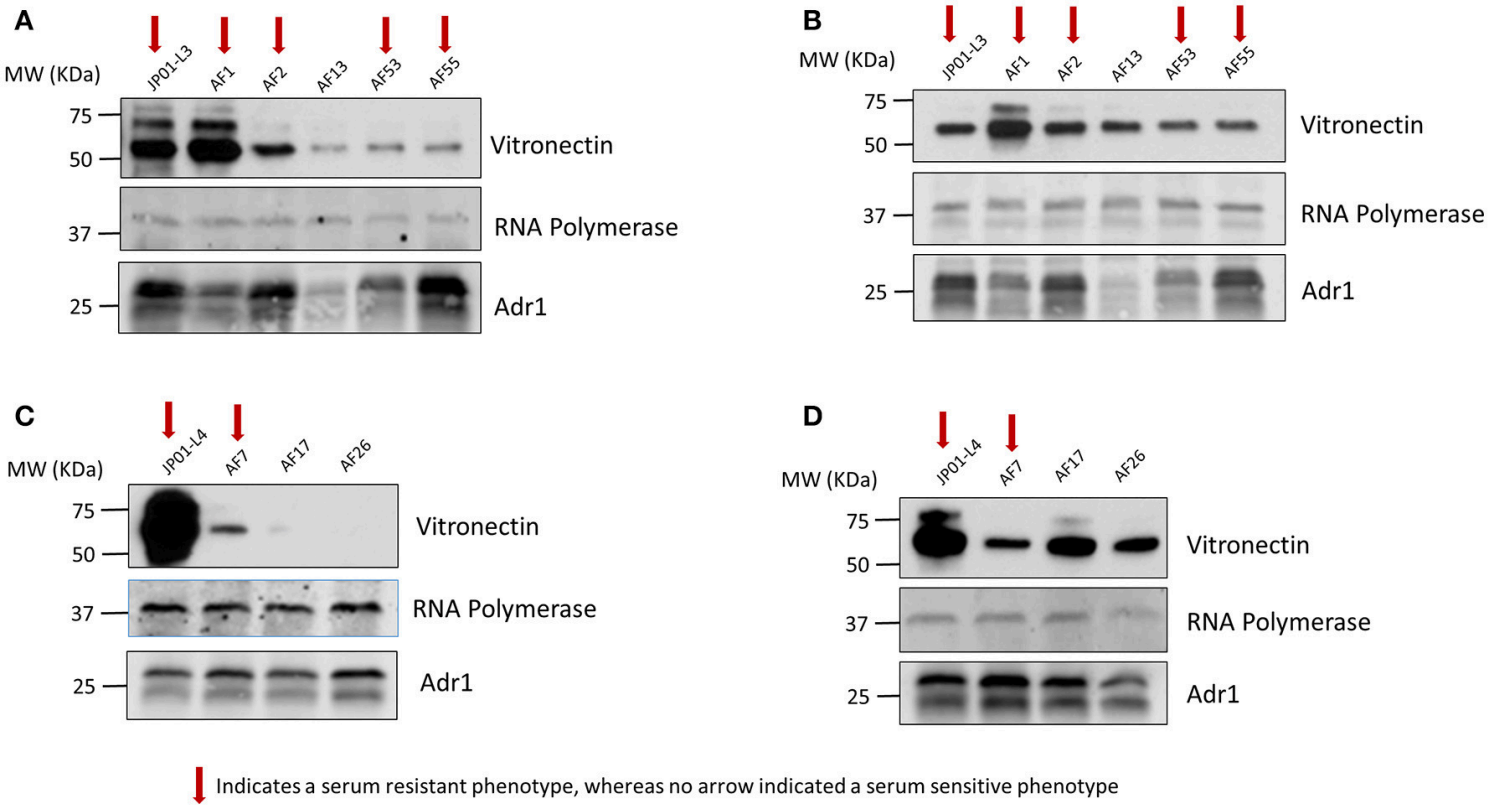
**Analysis of monomeric and multimeric vitronectin binding to loop 3 and 4 mutants. (A,C)** Western immunoblot analysis of multimeric vitronectin binding to *E. coli* expressing loop 3 or loop 4 Adr1 mutants with both serum resistant and serum-sensitive phenotypes. **(B,D)** Western immunoblot analysis of monomeric vitronectin binding to *E. coli* expressing loop 3 or loop 4 Adr1 mutants with both serum resistant and serum-sensitive phenotypes. Equal loading was verified using anti-*E. coli* RNA polymerase and Adr1 expression was verified using anti-Adr1. Data are representative of at least 3 replicates. Arrows depict Adr1 constructs that confer resistance to serum killing.

Vitronectin within the blood exists in both a monomeric and multimeric form. Consequently, Vn interactions that are responsible for Adr1-mediated serum resistance may in part be dependent on the form with which Adr1 interacts. Because the vitronectin multimer is the functionally active form we hypothesized that serum resistant mutants were able to bind to the multimeric form of Vn while serum-sensitive mutants were unable or significantly reduced in the ability to bind to the multimer. We also hypothesized that all mutants representing both serum-resistant and sensitive phenotypes would bind to the vitronectin monomer. To test our hypotheses, the aforementioned constructs were expressed at the outer membrane of *E. coli*. The bacteria were then incubated with monomeric and multimeric vitronectin and analyzed for the ability to bind to each form. The results of our experiment demonstrated that serum-resistant mutants bound to multimeric vitronectin (Figures 7A,C) whereas serum-sensitive mutants had a decreased ability to bind to the multimer. Conversely, all mutant regardless of phenotype were able to sufficiently bind to monomeric vitronectin (Figures 7B,D). These data indicate that electrostatic Adr1-Vn interactions are important for serum resistance and specifically serum resistance can be correlated with the ability of the bacteria to bind to the multimeric form of human vitronectin.

## Discussion

A previous report from this laboratory demonstrated that *R. conorii* binds vitronectin (Vn) to facilitate evasion of complement-mediated killing (Riley et al., 2014). Additionally, Adr1 loops 3 and 4 were sufficient to mediate the interaction with Vn. In the present study, we focused on elucidating the molecular determinants of the Adr1/vitronectin protein/protein interaction. Human vitronectin is a multifunctional human glycoprotein that is part of both the extracellular matrix and found in plasma. This protein plays a role in many biological functions including cell migration, tissue repair, and regulation of MAC formation by binding C5b-C7 complex and inhibition of C9 deposition on the surface of a cell or microbe (Singh et al., 2010b; da Silva et al., 2015). Vitronectin has a multi-domain structural arrangement that consists of an N-terminal somatomedian B domain which binds plasminogen activator inhibitor-1 and an RGD (arginine, glycine, and aspartic acid) domain that interacts with several integrins thereby aiding in attachment (Blom et al., 2009). Vitronectin also contains three heparin-binding domains and a C-terminal region with an unknown function, both of which have been shown to bind to surface proteins of bacterial pathogens (Blom et al., 2009; Singh et al., 2010a, 2011). Homologs to human vitronectin are found among many mammalian species including rabbits, mice, and cows. Interestingly, when these mammalian vitronectin proteins are compared there are regions of variability in both the N- and C-termini. Human vitronectin contains several unique residues within the C-terminal region, which may play an important role in protein-protein interactions (Leduc et al., 2009; da Silva et al., 2015). Because vitronectin is one of the major regulators of MAC formation, it plays a critical role in deposition of the MAC on bacterial pathogens. Gram-negative pathogens including *R. conorii, Legionella pneumophila, Neisseria meningitidis, Pseudomonas aeruginosa, M. caterrhalis*, and *H. influenzae* utilize vitronectin to protect against MAC killing (Hallstrom et al., 2006, 2009, 2016; Singh et al., 2010a; Riley et al., 2014). On the other hand, Gram-positive bacteria such as *S. pneumoniae* and *S. pyogenes* utilize vitronectin as a bridge to bind to and invade host cells (Leroy-Dudal et al., 2004; Singh et al., 2010b).

Our results indicate that the Adr1/Vn interaction is a heparin-independent, electrostatic interaction based on the ability of increasing concentrations of NaCl, but not heparin to inhibit the interaction between the two proteins. The ability of the recombinant peptide that contains a deletion within the C-terminal heparin binding domain (352–362) to bind to Adr1 when expression on the surface of *E. coli* further supports our conclusion that the Adr1/Vn interaction is heparin-independent. To our knowledge, this is the first demonstration of an outer-membrane protein in a Gram-negative bacterial pathogen that interacts with vitronectin in a heparin-independent, salt-sensitive manner. Heparin-dependent interactions, on the other hand, are well documented in Gram-negative pathogens (Hallstrom et al., 2016) and include proteins such as Ubiquitous surface protein A2 (UspA2) *of M. catarrhalis*, Haemophilus surface fibrils (Hsf) of *H. influenzae* type B, and Protein E (PE) and Protein F (PF) from non-typable *H. influenzae* (NTHi) (Hallstrom et al., 2006; Singh et al., 2010a, 2011; Su et al., 2013). Interestingly, a Gram-negative pathogen, *N. meningititis*, interacts with vitronectin via Meningococcal surface fibrils (Msf) in a heparin-independent manner; however, salt dependence has yet to be determined (Griffiths et al., 2011). Similarly to the Adr1/vitronectin interaction in *R. conorii*, the Gram-positive pathogen, *S. pneuomiae* interacts with vitronectin via Pneumococcal surface protein C (PspC) in a salt sensitive manner, signifying an electrostatic interaction. Increasing concentrations of salt competitively inhibited the ability of vitronectin to bind to PspC suggesting that the negatively charged amino acids in PspC mediate this interaction; however, individual amino acids responsible for this interaction have yet to be identified (Voss et al., 2013). The Adr1/Vn interaction is different from other bacterial/Vn interactions, and is likely unique to the Rickettsiales.

We utilized recombinant vitronectin peptides to elucidate the Vn region(s) required for association with Adr1. This region (amino acids 312–396) plays an important role in binding surface exposed proteins of both Gram-negative and Gram-positive bacterial pathogens (Singh et al., 2010a, 2011; Su et al., 2013; Voss et al., 2013; Hallstrom et al., 2016). Like many other bacterial pathogens, Adr1 from *R. conorii* was demonstrated to bind within the C-terminal region of vitronectin (amino acids 363–373), adjacent to the third heparin-binding domain. In contrast, many gram-negative and gram-positive bacteria including *S. pneumoniae, H. influenzae P. aeruginosa, Staphlococcus aureus, S. pyogenes* all of which have heparin-dependent interactions with vitronectin, bind within a domain (amino acids 352 and 374) containing a portion of the third heparin-binding domain (Hallstrom et al., 2006, 2016; Singh et al., 2011; Voss et al., 2013). *H. influenzae* also utilizes a heparin-mediated interaction with vitronectin, but the region required for binding to PE encompasses a longer sequence (amino acids 353–396) (Hallstrom et al., 2009, 2016).

A few other electrostatic protein-vitronectin interactions have been described. For example, the Gram-positive pathogen, *S. pneumoniae* utilizes PspC to bind vitronectin in a salt-sensitive manner. Although individual amino acids responsible were not identified, researchers suspect the negatively charged amino acids of the R domain in PspC mediate this interaction (Voss et al., 2013). In addition, the Gram-negative pathogen, *H. influenzae* binds vitronectin via PE in a heparin-dependent manner; however, two amino acids, leucine 85 and arginine 86 have been identified as critical for this interaction (Singh et al., 2011). Although leucine is an uncharged non-polar residue, arginine is positively charged and this charge could possibly contribute to the interaction. We determined that the Adr1/vitronectin interaction was an electrostatic interaction that was not mediated by a single charged residue. Instead, we observed that a substitution in the first two lysine residues in loops 3 and 4 caused a significant decrease the inability of *E. coli* expressing these proteins to evade the bactericidal effects of NHS. It is possible that these 2 amino acids in each loop independently create a critical initial interaction domain that is necessary for stable Adr1-vitronectin interactions and that without these two residues, Adr1 is not able to effectively mediate this protein-protein interaction. This hypothesis is further supported by the observation that perturbing the overall charge in Adr1 loop 3 had no deleterious effect on the ability of *E. coli* expressing this protein to survive serum-mediate killing.

Surprisingly, our results did not demonstrate a direct correlation between the ability to bind Vn and the ability to mediate survival in serum. Within human serum, vitronectin exists in both a monomeric and a multimeric form. The predominant form in plasma is a monomer and is also referred to as the “native” form (Stockmann et al., 1993). Multimeric vitronectin is found in small quantities, and consists of intra-molecular interactions between monomers (Stockmann et al., 1993). Previous studies examining vitronectin binding to outer-membrane proteins of various other pathogenic bacteria have looked at the native versus active form. The native form of vitronectin is folded, whereas the active form is conformationally altered and has an “open” structure, meaning that certain cryptic epitopes are available that are likely masked in the native form (Sa et al., 2010). Human serum is predicted to contain more than 7% of the activated form of vitronectin (Sa et al., 2010). Interestingly, the outer membrane protein, Opc of *N. meningitidis* exhibits preferential binding to the active form of vitronectin to mediate adhesion and invasion of brain cells (Sa et al., 2010). Conversely, *Yersinia pestis* utilizes two outer membrane proteins, Ail and Pla, to bind and proteolytically process the native form of vitronectin to facilitate resistance to complement mediated killing (Bartra et al., 2015). Upon further testing, we were demonstrated that serum-resistant Adr1 mutants bound both multimeric and monomeric Vn whereas serum-sensitive mutants bound to monomeric Vn, but were substantially impaired in their ability to bind to multimeric Vn. The data suggests that multimeric vitronectin mediates protection from complement-mediated killing. Therefore, vitronectin interactions that are responsible for Adr1-mediated serum resistance are dependent on the form with which Adr1 interacts. It is apparent that the critical Adr1-vitronectin interaction that contribute to *R. conorii* survival in serum is much more complex than originally thought.

Recent analysis of patients with confirmed cases of *R. conorii* infection demonstrated increased serum concentration of complement activation markers (Otterdal et al., 2016). The logical conclusion from those studies is that *R. conori* infection activates complement, potentially leading to release of inflammatory cytokines, and chemokines, and increases monocyte activation (Otterdal et al., 2016). While this data may appear to be contrary to our findings, a cursory glance at the complement cascade demonstrates that this is not the case. Herein and in previous publications, we describe rickettsial mechanisms for avoiding killing in serum. The Adr1/Vn interaction simply implies that, regardless of the state of complement activation, *R. conorii* is able to prevent activation, and deposition of the antibacterial terminal complement complex. As such, the findings of Otterdal et al. dovetail with our results, because *in vivo* complement activation indicates that *R. conorii* must avoid complement-mediated killing in order to establish (Riley et al., 2012, 2014).

*Neisseria meningitidis* is a human pathogen that causes increased morbidity and mortality worldwide as a result of sepsis and meningitis. Like *R. conorii, N. meningitidis* has the ability to bind to complement regulatory proteins in order to avoid complement mediated killing. Specifically, this bacteria binds Factor H via factor H-binding protein to avoid deposition of C3b and activation of the alternative arm of the complement system (Madico et al., 2006). When antibodies were created against this protein, the ability of the bacteria to bind Factor H was significantly decreased and as a result, the bacteria were not able to survive in serum (Madico et al., 2006). This discovery lead to the development of a preventative vaccine against multiple strains of *N. meningitidis* directed against factor H-binding protein (Seib et al., 2015; Gandhi et al., 2016). Herein, we demonstrate the molecular determinants of the interaction between *R. conorii* Adr1 and human Vn. The results gathered from this work further contribute to our understanding of the molecular mechanisms by which pathogenic rickettsial species establish successful infections in mammalian hosts. Elucidation of the interacting interface(s) in Adr1 and vitronectin will hopefully better guide the development of novel and efficacious anti-rickettsial therapies that specifically target this important protein-protein interaction.

## Author contributions

Conceived and designed experiments: AF, SR, JM, BS, and KR. Performed the experiments: AF and SR. Analyzed the data: AF, SR, and JM. Contributed reagents/materials/analysis tools: BS and KR. Wrote the paper: AF, SR, and JM.

## Funding

This work was supported in part by the National Institute of Allergy and Infectious Diseases of the National Institutes of Health under Award Numbers AI103912 and AI72606 (JM). This work was also supported by grants from the Alfred Österlund, the Anna and Edwin Berger, and the Greta and Johan Kock Foundations, the Swedish Medical Research Council (Grant K2015-57X-03163-43-4, http://www.vr.se), the Physiographical Society (Forssman's Foundation), and the Skåne County Council's Research and Development Foundation. We also acknowledge membership in and support from the Center for Experimental Infectious Disease Research at Louisiana State University (P20GM 103458). The content of this manuscript is solely the responsibility of the authors and does not necessarily represent the official views of the National Institutes of Health.

### Conflict of interest statement

The authors declare that the research was conducted in the absence of any commercial or financial relationships that could be construed as a potential conflict of interest.

## Supplementary material

Supplemental Figure 1 **Densitometric Analysis of Vitronectin binding. (A,B)** Densitometric analysis of vitronectin to *E. coli* RNA polymerase signal when Adr1 is expressed at the surface of *E. coli* and exposed to vitronectin in the presence of increasing concentrations of heparin or salt. **(C,D)** Densitometric analysis of vitronectin to OmpB signal when vitronectin is exposed to *R. conorii* in the presence of increasing concentrations of heparin or salt. A one-way ANOVA with a Newman-Keuls *post hoc* test was performed on all data. ^*^Represent a *p* ≤ 0.05 and is considered significant. ns represents no significance.

Supplemental Figure 2 **Expression of Adr1 loop 3 mutants in ***E. coli*** BL21(DE3)**. Western immunoblot analysis of whole cell lysates for each mutant constructed within loop 3 using anti-Adr1.

Supplemental Figure 3 **Expression of Adr1 loop 4 mutants in ***E. coli*** BL21(DE3)**. Western immunoblot analysis of whole cell lysates for each mutant constructed within loop 4 using anti-Adr1.

Supplemental Figure 4 **Confirmation of expression of mutant Adr1 proteins at the outer-membrane of ***E. coli*****. Western immune blot analysis of whole cell lysates (WCL), sarkosyl soluable/inner membrane proteins (IM) and outer membrane fractions (OM). An Adr1 derivative containing only loop 4 (JP01-L4) was used as a positive control and the empty vector (pET22b) was used as the negative control. Representative constructs of serum resistant (AF8) and serum-sensitive (AF17) and phenotypes are presented.

Supplemental Table 1 **Table of primers used for site-directed mutagenesis**. Forward, reverse and screening primers are listed for each lysine to alanine mutant that was created in this body of work.
