# Supplementary figures and images for: The Rickettsia conorii Adr1 Interacts with the C-Terminus of Human Vitronectin in a Salt-Sensitive Manner
Source: Front Cell Infect Microbiol. 2017 Mar 1;7:61. doi: 10.3389/fcimb.2017.00061 (PMC5331051; doi:10.3389/fcimb.2017.00061)

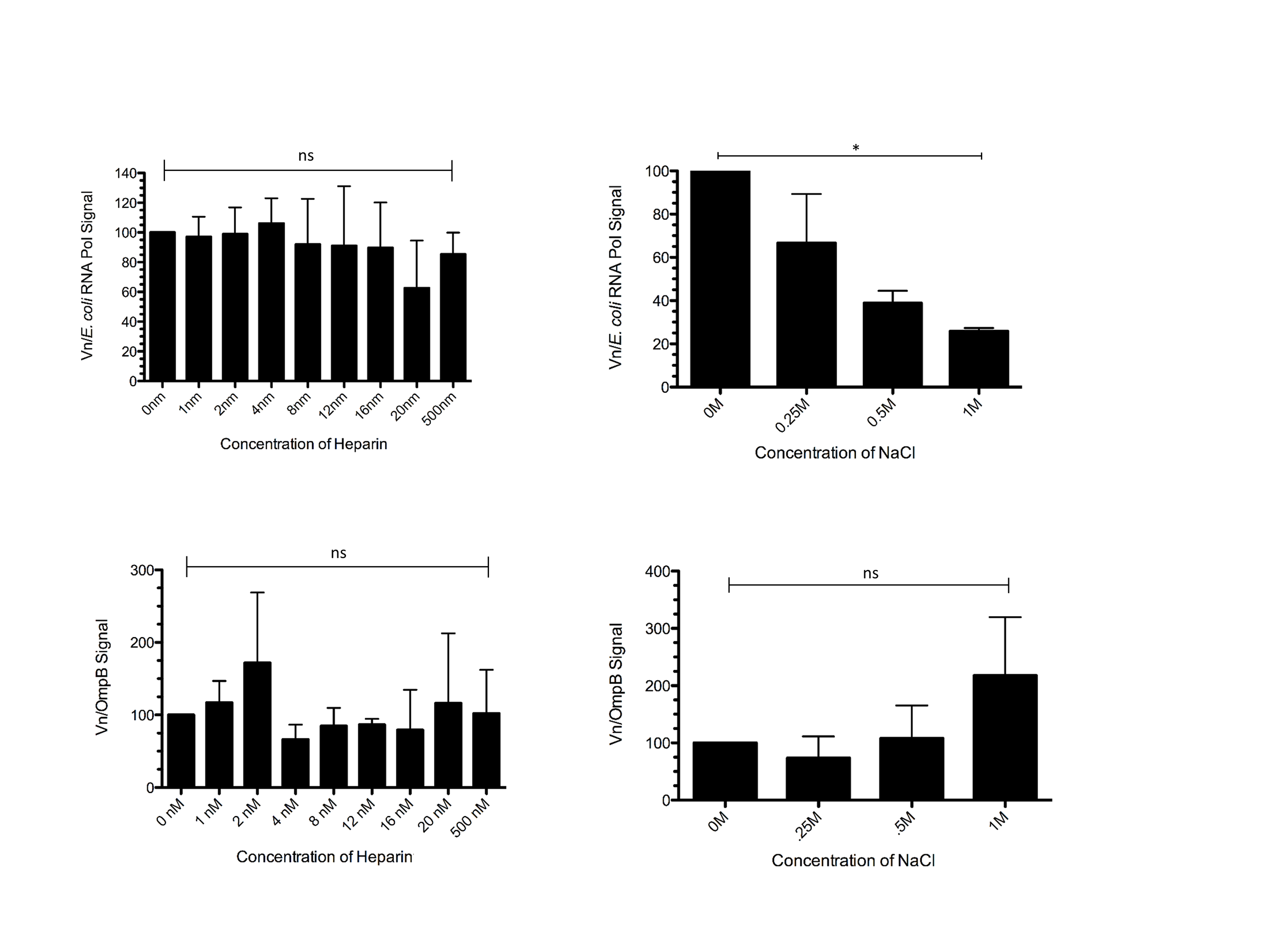

Supplement: Supplemental Figure 1 — Densitometric Analysis of Vitronectin binding. (A,B) Densitometric analysis of vitronectin to E. coli RNA polymerase signal when Adr1 is expressed at the surface of E. coli and exposed to vitronectin in the presence of increasing concentrations of heparin or salt. (C,D) Densitometric analysis of vitronectin to OmpB signal when vitronectin is exposed to R. conorii in the presence of increasing concentrations of heparin or salt. A one-way ANOVA with a Newman-Keuls post hoc test was performed on all data. *Represent a p ≤ 0.05 and is considered significant. ns represents no significance. [file Image1.TIF]

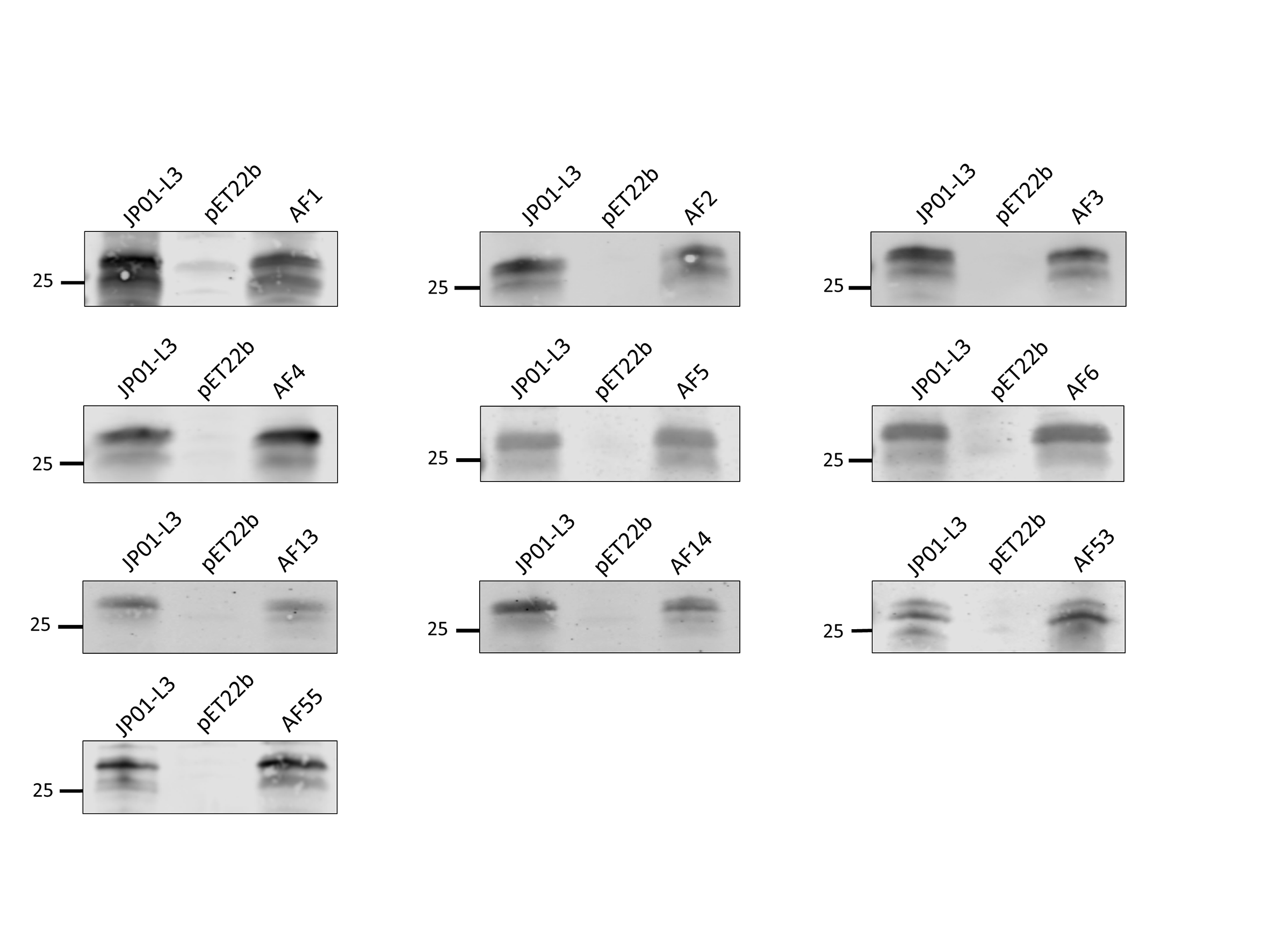

Supplement: Supplemental Figure 2 — Expression of Adr1 loop 3 mutants in E. coli BL21(DE3). Western immunoblot analysis of whole cell lysates for each mutant constructed within loop 3 using anti-Adr1. [file Image2.TIF]

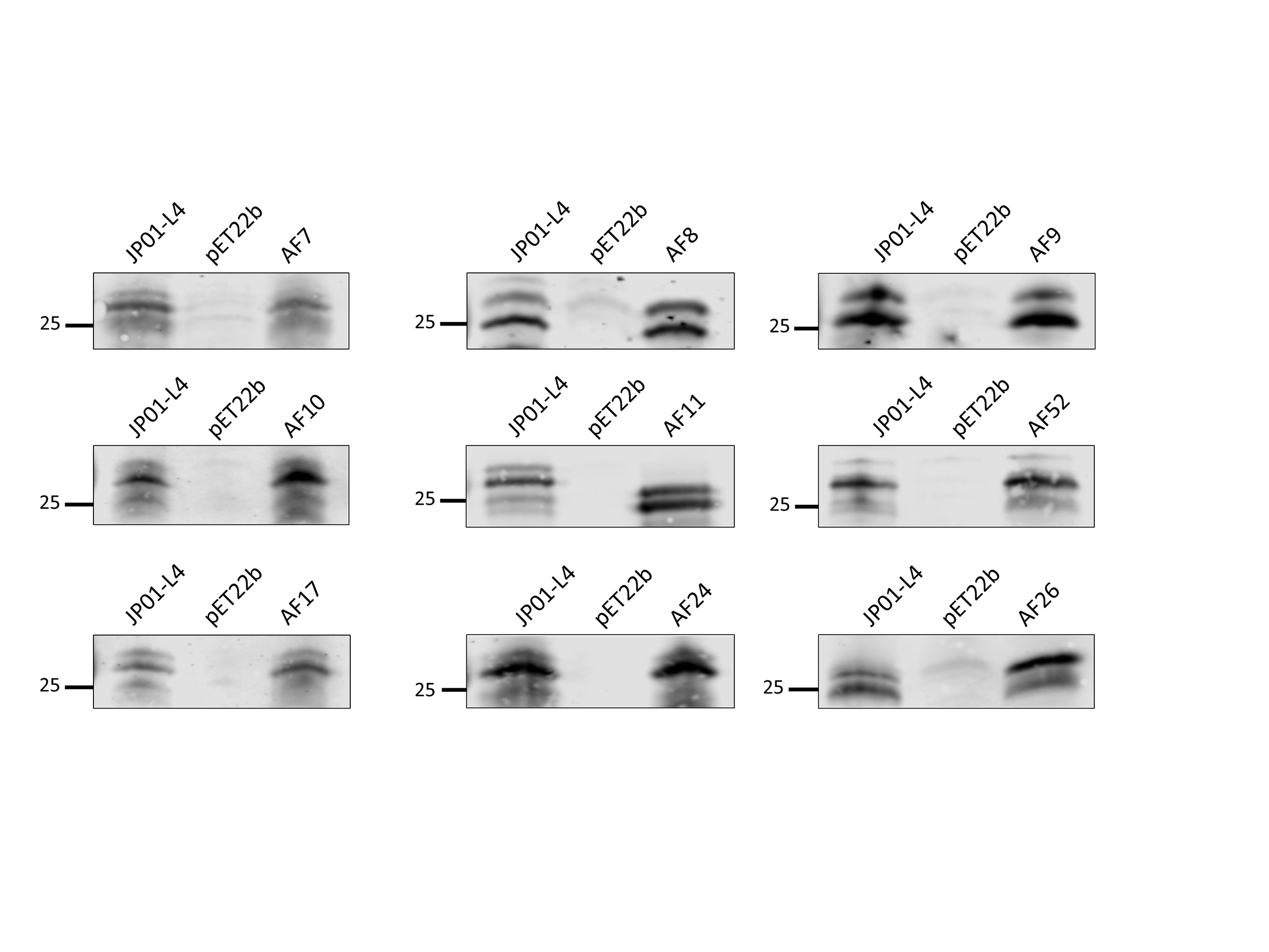

Supplement: Supplemental Figure 3 — Expression of Adr1 loop 4 mutants in E. coli BL21(DE3). Western immunoblot analysis of whole cell lysates for each mutant constructed within loop 4 using anti-Adr1. [file Image3.TIF]

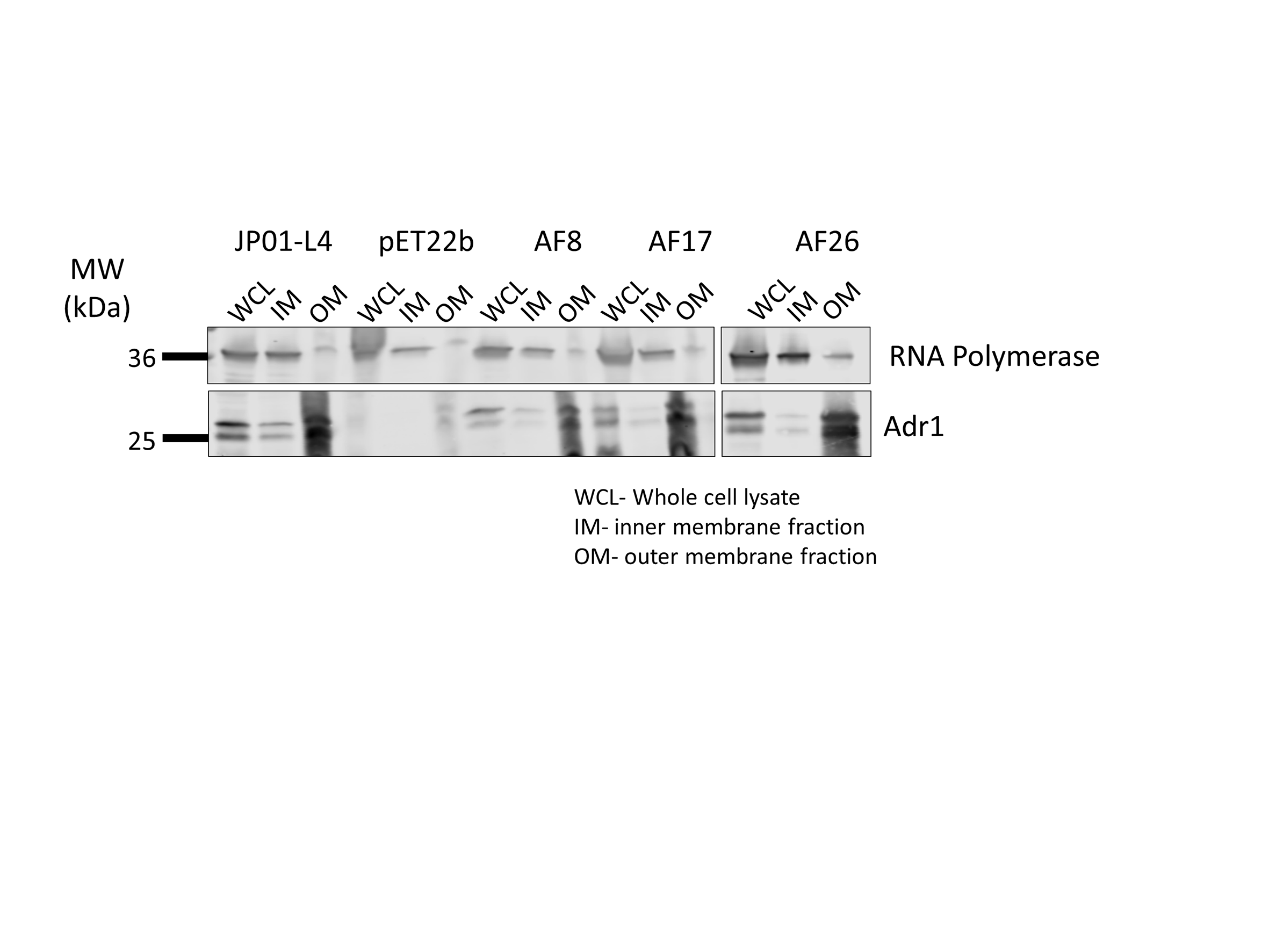

Supplement: Supplemental Figure 4 — Confirmation of expression of mutant Adr1 proteins at the outer-membrane of E. coli. Western immune blot analysis of whole cell lysates (WCL), sarkosyl soluable/inner membrane proteins (IM) and outer membrane fractions (OM). An Adr1 derivative containing only loop 4 (JP01-L4) was used as a positive control and the empty vector (pET22b) was used as the negative control. Representative constructs of serum resistant (AF8) and serum-sensitive (AF17) and phenotypes are presented. [file Image4.TIF]
